# A Novel Approach for Treatment of an Unusual Presentation of Radicular Cysts Using Autologous Periosteum and Platelet-Rich Fibrin in Combination with Demineralized Freeze-Dried Bone Allograft

**DOI:** 10.1155/2013/893791

**Published:** 2013-08-01

**Authors:** Veena A. Patil, Manthan H. Desai, Veerendra S. Patil, Hanisha Reddy Kaveti, Kiran Kumar Ganji, Prasanna M. Danappanavar

**Affiliations:** ^1^Department of Periodontology, HKES's S.Nijalingappa Institute of Dental Sciences and Research, Sedam Road, Gulbarga, Karnataka 585105, India; ^2^Department of Endodontics, HKES's S.Nijalingappa Institute of Dental Sciences and Research, Sedam Road, Gulbarga, Karnataka 585105, India; ^3^Department of Periodontics, Sharad Pawar Dental College & Hospital, Sawangi Meghe Wardha, Maharashtra 442004, India; ^4^Department of Oral and Maxillofacial Pathology, MNR Dental College & Hospital, Sangareddy, Medak, Andhra Pradesh 502294, India

## Abstract

Radicular cysts are the most common cystic lesions affecting the jaws. They are most commonly found at the apices of the involved teeth. This condition is usually asymptomatic but can result in a slow-growth tumefaction in the affected region. The following case report presents the successful treatment of radicular cysts using autologous periosteum and platelet-rich fibrin with demineralized freeze-dried bone allograft.

## 1. Introduction

Radicular cysts are the most common (52%–68%) cystic lesions affecting the jaw [1]. They are commonly found at the apices of involved teeth and sometimes lateral to accessory root canals. They are a direct sequel of chronic periapical infection [1]. Most of them are asymptomatic and are discovered when periapical radiographs are taken of teeth with nonvital pulps. Patient often complains of slowly enlarging swellings. Radiographically, most radicular cysts appear as round or pear shaped unilocular radiolucent lesions in the periapical region. The cyst may displace adjacent teeth or cause mild root resorption [2].

The following case report presents the successful treatment of radicular cysts using autologous periosteum and platelet-rich fibrin (PRF) with demineralized freeze-dried bone allograft (DFDBA).

## 2. Case Report

A 17-year-old female patient reported to the Department of Periodontics, HKES's S.Nijalingappa Institute of Dental Sciences and Research, Gulbarga, India, with a chief complaint of pain, swelling ongoing and pus discharge in the lower anterior region since two months. Past history revealed trauma in the lower anterior region 5 years ago with recurrent swelling and pus discharge.

**Figure 1:**
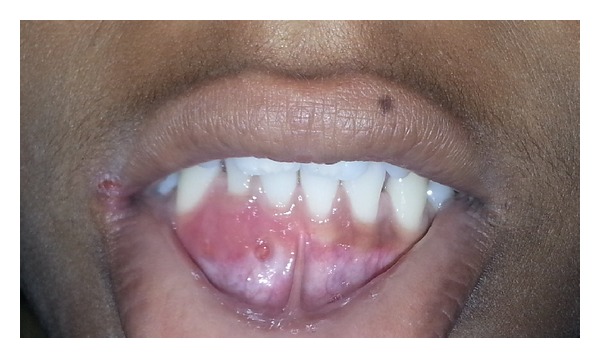
Preoperative view of the lesion.

**Figure 2:**
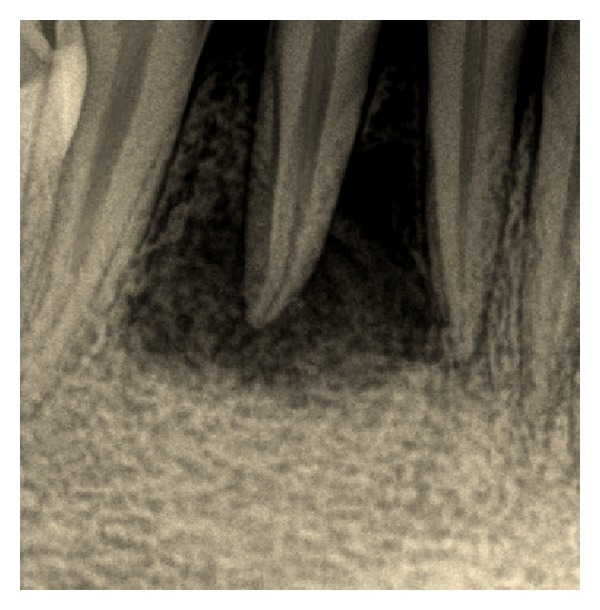
Preoperative radiograph.

On intraoral examination, inflamed and swollen gingiva was seen in relation to 41, 42, and 43 (FDI notation). A draining fistula was seen on the labial aspect in relation to 41 (Figure 1). 42 had grade I mobility, whereas no mobility was noticed with 31, 41, and 43. Pulp vitality test was negative with 41, 42, and 43, while adjacent teeth showed normal response. Periodontal probing depth was ≤3 mm for concerned teeth, and no clinical attachment loss was seen. They were also painless on vertical percussion. On radiographic examination, two radiolucent areas of size approximately 2 × 2 mm were seen in relation to 41, 42, and 43 (Figure 2). No root resorption was seen.

The treatment plan comprised of endodontic treatment of nonvital teeth followed by surgical enucleation of cystic lesions if necessary. Treatment plan was explained to the patient, and a written informed consent was obtained. In the same visit, root canal treatment was started under rubber dam application followed by working length determination. After complete biomechanical preparation, 2% chlorhexidine gluconate was used as an irrigant and intracanal medicament. In the subsequent visits, root canal treatment was completed. Persistent pus discharge was observed at 3 months after endodontic treatment, and surgical enucleation was planned.

The procedure is as follows: local anesthesia was administered, crevicular incisions were given, and a full thickness mucoperiosteal flap from 41 to 43 and a split thickness flap in 31, 32 region were reflected. The area was degranulated revealing two small perforations of the buccal cortical plate in the regions of 41 to 43 of size 1 × 1 × 1 mm. The remaining buccal cortical covering was carefully removed with rotary and hand instruments to expose the rest of the lesions of size 3 × 3 × 2 mm.

**Figure 3:**
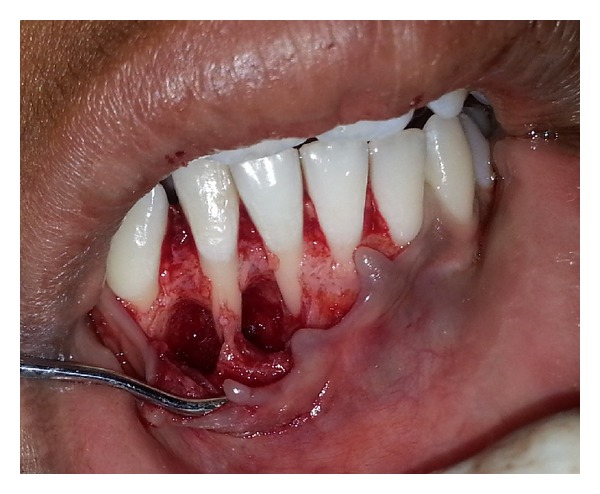
Debridement of the lesions.

**Figure 4:**
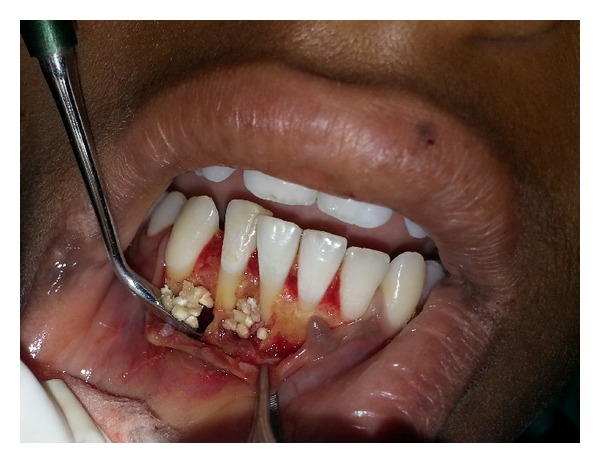
DFDBA graft placement.

**Figure 5:**
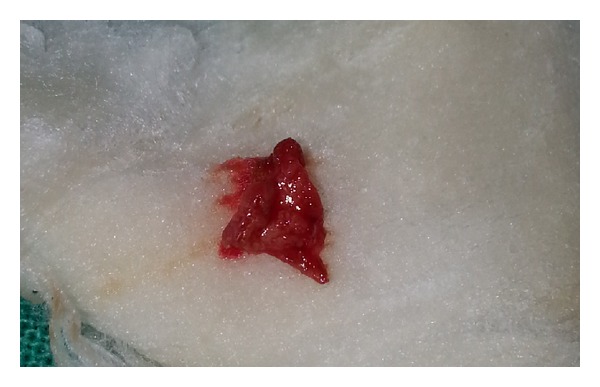
Harvested autologous periosteum.

**Figure 6:**
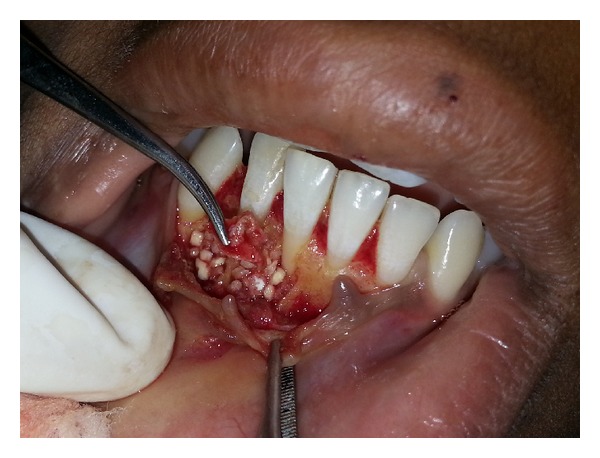
Autologous periosteum placed as a barrier membrane.

**Figure 7:**
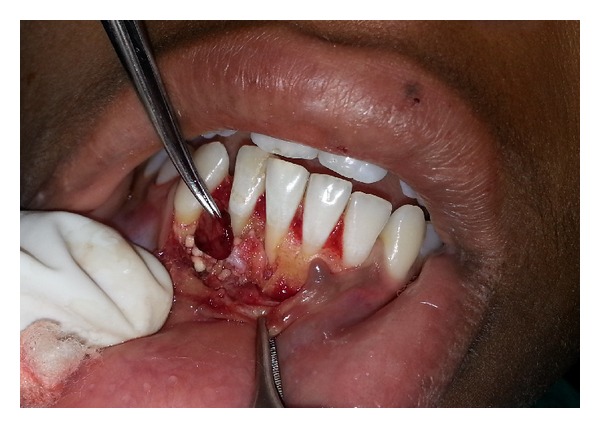
PRF placed as a barrier membrane.

Fragmented pieces of the lesion were freed from the bone, and complete curettage of the cystic lesions was done (Figure 3). The cystic cavities were thoroughly irrigated, and root biomodification of involved teeth was done using tetracycline. DFDBA was mixed with sterile saline solution and grafted in an attempt to close the defect via osteoconduction (Figure 4). Autologous healthy periosteum was harvested from the 31-32 region (Figure 5), and PRF was prepared from the patient's blood, as described by Choukroun et al. [3]. The lesion was covered with periosteum, over which PRF was placed as a second layer of barrier membrane covering the graft (Figures 6 and 7).

**Figure 8:**
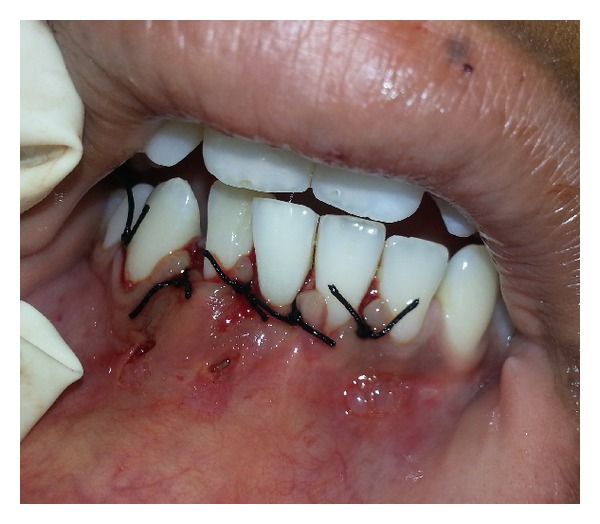
Sutured flap with 3-0 silk suture.

The flap was coronally advanced and closed with interrupted sutures using 3-0 black braided silk (Figure 8). A periodontal dressing was applied at the surgical site. The patient was prescribed amoxicillin 500 mg TID and diclofenac sodium 50 mg TID both for 5 days with 0.12% chlorhexidine gluconate rinse BD for 7 days. Patient was asked to report after a week for suture removal, and the curetted tissue was submitted for histopathological examination. The patient returned for the postoperative visit, and the healing was uneventful.

**Figure 9:**
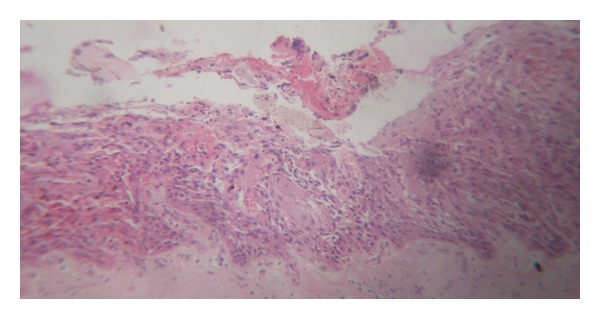
Histopathology of excised cyst.

**Figure 10:**
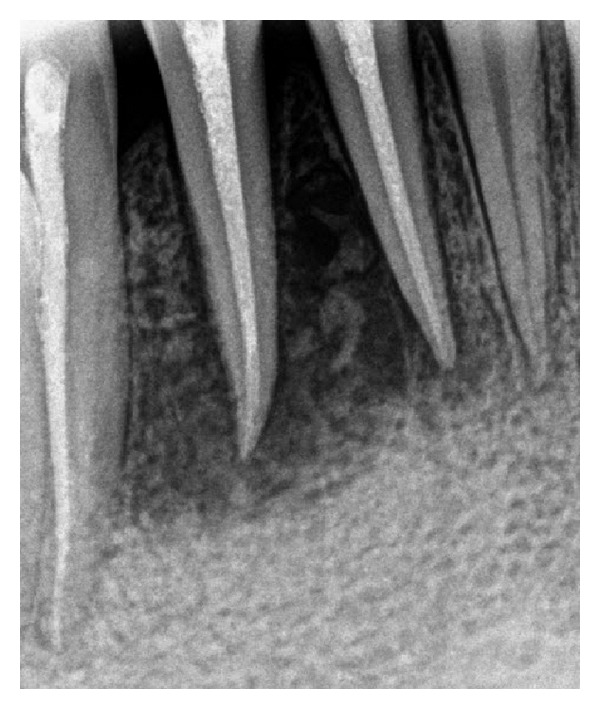
6 months postoperative radiograph.

**Figure 11:**
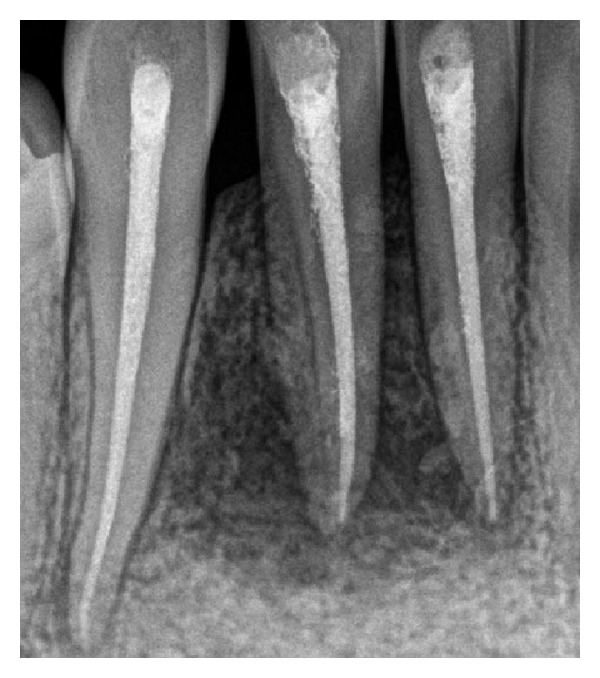
9 months postoperative radiograph.

**Figure 12:**
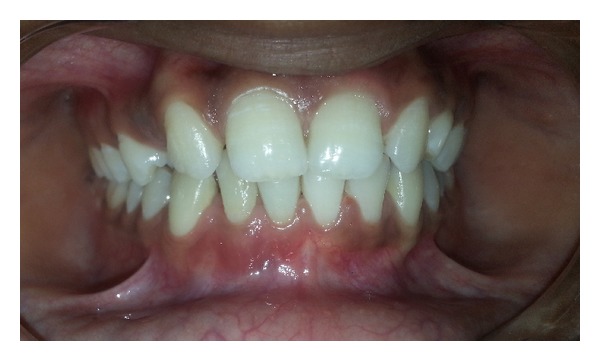
Healing at 9 months after operation.

Histopathology revealed the presence of a varying thickness of epithelium with fibrocellular connective stroma. The epithelium was disrupted with infiltration of chronic inflammatory cells along with vacuolations within the epithelium. Connective tissue showed dense infiltration of lymphocytes and plasma cells with few macrophages (Figure 9). A diagnosis of radicular cyst was given. Patient was followed up for 9 months. Radiograph at 6 months shows a healing lesion (Figure 10). A subsequent radiograph 9 months after operation (Figure 11) reveals increased radiopacity where the bone graft was placed, and no evidence of recurrence of the lesion was seen (Figure 12).

## 3. Discussion 

A radicular cyst is an odontogenic cyst of inflammatory origin preceded by a chronic periapical granuloma and stimulation of cell rests of Malassez found in the periodontal membrane. The pathogenesis of radicular cysts comprises of three distinct phases: the phase of initiation, the phase of cyst formation, and the phase of enlargement [4]. The initial swellings of these radicular cysts are usually bony hard, but as they increase in size, the covering bone may become very thin despite initial subperiosteal bone deposition. With progressive bone resorption, the swellings exhibit “egg shell crackling.” The associated teeth are always nonvital and may show discoloration. Although the associated teeth usually show no root resorption, there may be smooth resorption of root apices. When cysts are intact, cyst cavities may be filled with brown- or straw-colored fluid, giving them a shimmering gold appearance [4]. Radicular cysts are inflammatory lesions leading to bone resorption and can reach great dimensions and become symptomatic when infected or with great size due to nerve compression.

The main cause of failure of endodontic treatment is generally accepted to be the continuing presence of microorganisms in the root canal system that have either resisted treatment or have reinfected the root canal system. *E. faecalis* was the most frequently found microbe in such cases [5]. Chlorhexidine gluconate has been proposed for use both as an irrigant and as a medicament especially in endodontic retreatment. As a medicament, it is more effective than calcium hydroxide in eliminating *E. faecalis* infection inside dentinal tubules [6]. As an irrigant, it appears as effective or superior to sodium hypochlorite in the elimination of *E. faecalis* [7].

The adult human periosteum is highly vascular and is known to contain fibroblasts, osteoblasts, and stem cells. Skoog [8] subsequently introduced the use of periosteal flaps for closure of maxillary cleft defects in humans; he reported the presence of new bone in cleft defects within 3–6 months following surgery. Furthermore, animal studies have reported heterotopic ossification in different organs after implantation of free periosteal grafts [9, 10]. In all age groups, the cells of the periosteum retain the ability to differentiate into various cells. [11]. On the basis of these observations, it can be hypothesized that the periosteal membrane can contribute to the stimulation of new bone formation and has an immense potential for regeneration.

PRF belongs to the new generation of platelet concentrates with simplified processing. PRF contains a variety of growth factors, which enhance healing by increasing angiogenesis and matrix biosynthesis [12]. The immense osteoinductive capability of DFDBA is well described in the periodontal literature [13].

The treatments of these cysts are still under discussion, and many professionals opt for a conservative treatment by means of endodontic technique [14]. However, in large or nonhealing lesions, the endodontic treatment alone is not efficient, and surgical treatments like marsupialization or enucleation should be considered [15]. In this case, surgical enucleation was preferred and was performed uneventfully.

## 4. Conclusion 

To conclude, a radicular cyst is a common condition found in the oral cavity. However, it usually goes unnoticed and rarely exceeds the palpable dimension. This case report illustrates the successful management of a radicular cyst with enucleation and endodontic treatment. The use of autologous periosteum and PRF has a promising future in periodontal regeneration.
